# Navigating Unmountable Media with the Digital Forensics XML File System

**DOI:** 10.2218/ijdc.v12i2.581

**Published:** 2017

**Authors:** Alex Nelson, Alexandra Chassanoff, Alexandra Holloway

**Affiliations:** National Institute of Standards and Technology; Massachusetts Institute of Technology Libraries; Jet Propulsion Laboratory / California Institute of Technology

## Abstract

Some computer storage is non-navigable by current general-purpose computers. This could be because of obsolete interface software, or a more specialized storage system lacking widespread support. These storage systems may contain artifacts of great cultural, historical, or technical significance, but implementing compatible interfaces may be beyond available resources.

We developed the DFXML File System (DFXMLFS) to enable navigation of arbitrary storage systems that fulfill a minimum feature set of the POSIX file system standard. Our approach advocates for a two-step workflow that separates parsing the storage’s file system structures from navigating the storage like a contemporary file system, including file contents. The parse extracts essential file system metadata, serializing to Digital Forensics XML for later consumption as a read-only file system.

## Introduction

^1^Since their inception, computer storage systems have had a user interface with a fixed primitive set, including the named file that references addresses for file content. However, despite this stable conceptual base, accessing files on antiquated storage systems is not always possible. Hardware and software age and fall out of support as operating system design progresses, making some storage uninterpretable. Further, some specialized storage systems—*e.g*. game consoles (Nelson, Steggall, and Long, 2014)—decline to implement widespread support for commercial or consumer operating systems.

We address in this paper the problem of treating the data of an outdated or uncommon computer storage system like the data of a contemporary file system. Contemporary computer operating systems have code to attach *(i.e.* “mount”) current file systems to their storage namespace, enabling users and programs to walk the file system, and read and write files. If a file system isn’t supported in the operating system, but access to its files and their metadata are desired, then software must be developed. Typically, that software bundles logic to parse the storage with other interfacing software, from custom navigation shells^2^ to full original file system kernel modules. Unfortunately, in these approaches, functionality can be hindered or prevented due to ongoing maintenance requirements and lack of implementation resources.

We present a file system called the Digital Forensics XML File System (“DFXMLFS”), and an accompanying workflow that normalizes access to storage systems, requiring only the development of a storage system parser. The key element of our approach is separating file system *parsing* from file system *interfacing.* We provide background on why this approach is possible, historically and technologically. We describe scenarios in which this practice can be beneficial and critical. Finally, we characterize essential components and provided examples to assist motivated developers with the broader goal of enabling access to outdated or uncommon storage systems as economically as possible.

## Background

File systems have grown feature sets from a core concept of hierarchical file organization. Some examples of integrated features include full-disk encryption (e.g. APFS for macOS, 2016), data and metadata checksumming (e.g. ZFS for OpenSolaris, 2005), live system repair (e.g. ZFS), and quota groups (e.g. Btrfs). Yet for the typical computer user, interaction with the file system and corresponding user needs have not evolved much beyond search and access capabilities. The POSIX family of standards from the late 1980s defined a consistent user interface in terms of command-line functionality, including the way the user navigates the file system and the way that the file system is displayed (IEEE, 1988). Even with the popularization of graphical windowed environments, the feature set of file systems—particularly what is visible to the user—has remained largely the same (Reimer, 2008). Indeed, file system user interfaces have remained largely the same in the past 30 years or more (S. Ames, Maltzahn, and Miller, 2008). Users typically have little knowledge or insight into file system level features such as journaling or data de-duplication; even with those advanced features, users may rely on file listings alone for organization and access. As we increasingly make and accumulate our own streams of data on a variety of media, users may wish to examine specific file system features for different purposes and through a variety of methods. Digital archivists and forensic analysts share some needs for metadata features that have remained stable since near the beginning of file systems. For example, an archivist may wish to gather contextual information about the modification, access, and change (MAC) times of a file system to ensure provenance (Woods, Lee, and Misra, 2013). A scholar may also wish to recover a file that was previously thought to be deleted (Kirschenbaum, Ovenden, Redwine, and Donahue, 2010).

DFXMLFS is a FUSE (Szeredi, 2006) file system that leverages DFXML (S. Garfinkel, 2012) to separate the file system parsing and interface software. DFXMLFS is largely possible because of file systems’ long-lasting interface stability. It presents a navigable interface to robust file system metadata while also complying with a broad common denominator in contemporary storage interface requirements, such as adhering to the navigating and file-reading components of the POSIX interface (IEEE, 1988). The end result is the ability to navigate any hierarchical storage medium that has previously implemented the POSIX interface, even if the medium’s last living interface software lost support decades ago. Critically, the work required for this functionality restoration is significantly less per file system type, in comparison with updating an original implementation most likely tied to an also-obsolesced operating system. It joins two technologies to enable storage system access and demonstrates the benefits of adopting an in-common practice, following an in-common language specification.

### FUSE

Filesystem in Userspace (FUSE) (Szeredi, 2006) is a library that offers an alternative system for developing file systems. FUSE moves file system development from implementing a kernel module to instead writing userspace functions. This has aided the prototyping of many file system designs that were either experimental or more fit for userspace operations. The related work section will discuss some example alternatives to traditional hierarchical file navigation.

### DFXML

Digital Forensics XML (DFXML) provides a plaintext language for viewing file system artifacts. DFXML stores storage-forensic tool output, enabling capture and representation of file system metadata to ensure provenance, authenticity, and integrity of storage media (Woods, Chassanoff, and Lee, 2013). One objective behind its development was to automate some components of storage analysis that could frequently be done without access to the disk image (S. L. Garfinkel, 2009; S. Garfinkel, 2012). For instance, timeline analysis and file signature recognition only need limited metadata from the storage system (file timestamps and content checksums), and only once.

DFXML is a sufficient manifest to enact full storage navigation and file extraction without needing to re-parse on-disk structures after the XML is generated. DFXML provides a vocabulary that supports the data necessary to create an in-memory file system tree, including file properties like name, path, size, times, data addresses, and checksums.

This is not the full extent of file properties that DFXML can capture, but it is sufficient for most file system navigation and content viewing needs. The Digital Forensics XML schema^3^ provides full documentation on the structure and other metadata fields in a DFXML file.

## Related Work

### FUSE & Alternate Approaches to Navigating Files

Some projects have taken an attribute-based approach to navigating files, including hierarchies like metadata query construction. For example, a virtual directory entitled “mp3” could filter an audio file collection down to MP3s, and another directory under that titled “bitrate_320kbps” further filters to select a certain bit rate. Folder hierarchies can also be used to organize parameterized experiment results, using directories to note parameter values used (Strong, Jones, Parker-Wood, Holloway, and Long, 2011). This approach to navigating file sets by attributes carries several challenges in metadata identification and indexing (A. Ames et al., 2005; Parker-Wood, Long, Miller, Rigaux, and Isaacson, 2014), but can offer a useful alternative to fixed directory hierarchies when considering files with rich and consistent metadata.

It is possible to use FUSE as more of an intermediary layer to access another file system. The SSH File System (SSHFS) acts in the same way an NFS client does, “mounting” a remote system’s directory locally, but only requiring SSH access instead of an NFS server process to be actively exporting a share.

### Normalizing Storage System Interfaces

Recovery of computer storage contents has several different levels of challenges. One challenge Nelson et al. (2014) identified in inspection of an uncommon file system was that several of the analysis tools employed for analyzing *file systems* were designed assuming that the input disk image was expected to be an image of the *disk partition* containing the file system. Unfortunately, storage analysis in forensic and curation processes typically begin with images of entire disks, which have a partition management format that further points to disk partitions. The game console file system Nelson *et al.* inspected also had a custom partition management system. They applied a subset of the same practice as suggested in this paper, but for the purpose of accessing file systems: a forensic tool would inspect the partition management system, and then present a FUSE-based file system, *UPartsFS,* that offered each disk partition as its own virtual file. This relieves file system analysis tools from needing additional logic to handle partitioning systems as well.

The present implementation of *UPartsFS* relies on a version of The SleuthKit (Carrier, 2003) modified to analyze this uncommon storage. *UPartsFS* could be modified to use an XML representation of partition tables, which would remove its reliance on a customized version of another tool’s code base. Then other tools can be used to recognize disk images, and output appropriate partition type and size metadata *(e.g.* as DFXML). Switching to an XML representation for partition data would help integrate research from file type identification (Underwood, 2013).

### Current Access Strategies

DFXMLFS offers one strategy to enable access to an uncommon storage medium that is unsupported by current operating systems. There are several other strategies also available today, listed in Table 1 in mostly decreasing order of implementation difficulty.

DFXMLFS provides an alternative option, requiring a smaller base of programming experience. A forensic tool can be developed from scratch (or adapted from available-source options) to parse a storage system and serialize the storage data structures as a metadata manifest in DFXML, instead of implementing a user interface. DFXMLFS then handles joining the tool’s output with a kernel’s file system interface, enabling standard file-listing and directory-walking interactions by mounting the DFXML file like any read-only medium. If data addresses or file extraction commands are included in the XML, the original disk image can also be provided alongside the DFXML to enable read-only file extraction. However, only some DFXML generation strategies can yield that level of supporting metadata.

## DFXMLFS Usage Workflow

The objective of DFXMLFS is to normalize navigation of arbitrary hierarchical file systems. DFXMLFS usage follows a *parse-serialize-transport-deserialize* workflow. Our workflow serializes file systems to DFXML as an intermediary, text-based format, and uses the FUSE framework to deserialize that text into a modern-acting file system in a later process—even on a separate system. The DFXMLFS program—the implemented FUSE interface—handles deserialization and user presentation. Usage of DFXMLFS is still a “Workflow,” because DFXMLFS does not come built in with a universal parser for all file systems. Each file system requires special-case handling, in many cases by whomever finds themselves with an uncommon storage system in hand. What is required most of that analyst is locating or developing the first of the workflow steps: A parser.

### Storage parsing

A *file system parser* is a program that populates data structures of a file system API— nominally, the POSIX Virtual File System interface. At other points in this paper, instead of *file systems, storage systems* are referenced. A *storage system* is meant to entail some storage device or image that contains one or more file systems, organized by a *partition system.* A *storage system parser* has the additional step of parsing partition systems before parsing file systems.

A file system parse will typically result in finding at least the following information:

- File size
- Timestamps, such as last modification, last metadata change, last access, and creation
- File path from the root of the file system

Normally, the kernel would also want some type of list of on-disk addresses of data blocks that store file and directory contents. However, this is not strictly necessary. A running file system process only needs to be able to respond sanely to a read(dest_buffer, offset, length, file_handle) system call, which only requires bytes yielded from a byte stream.

### Serialization

**Figure 2.**
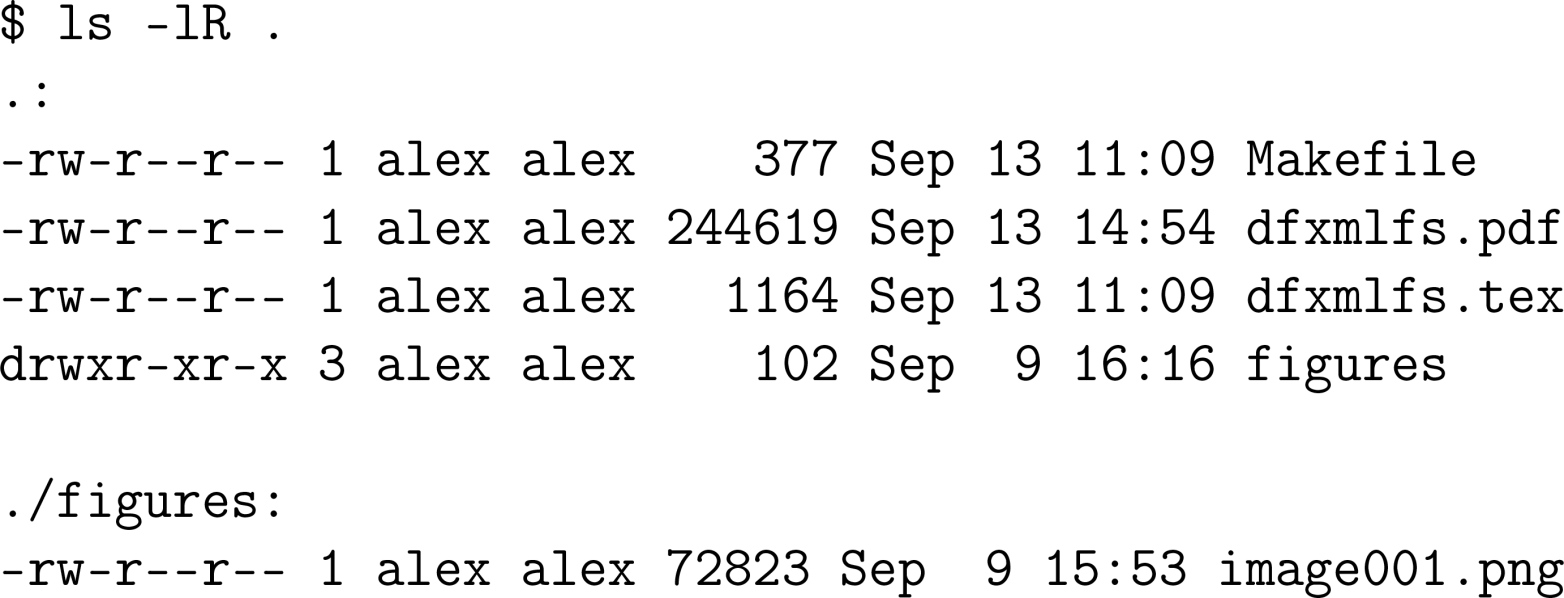
A recursive directory listing.

Navigating serialized metadata may be best illustrated by observing directory listing information. For example, Figure 2 shows a recursive directory listing in a modern, POSIX-based file system. It is possible to construct a navigable file system from this information: the listing shows there are two directories with four named files, and their modification times. However, this text listing is not sufficient *to view* the file contents. To meet that objective, we turn to DFXML to represent usual file metadata users see, as in ls output, and data location metadata as well.

### Deployment

DFXMLFS is implemented and currently available. What is left to the interested digital curator or storage analyst is parsing and serializing the storage, either by finding a DFXML generator or developing one. A later section describes available generators, which can serve as working examples if code meeting the analysis objective is not available. First, we describe the implementation of DFXMLFS, so the user may understand what is needed to enable normal storage interaction.

## DFXMLFS Implementation

DFXMLFS joins two technologies that have focused on simplifying file system design and analytics, in order to implement read() and other calls that comprise a file system interface. DFXML’s Python support includes a library of Objects^4^, which read and write XML documents and provide an object-oriented programming interface. DFXMLFS implements file system functions in the Python FUSE bindings (Szeredi, Henk, Delafond, James, and Epler, 2004) that are sufficient to expose a read-only file system to the user.

Figure 2 showed that with inode data extractable with the stat command, one can populate all but file contents for an entire directory hierarchy. This information is often exposed by tools that implement a navigation shell. The more difficult challenge is in presenting file content. A DFXML generator has to provide one of two things:

1. byte_runs elements for each file’s content.
2. A command to use a tool to extract file content into a cache, to which the FUSE bindings can pass reading operations with regular system calls.

There is a trade-off in the choice made for file extraction strategy. If individual extraction commands are embedded in the DFXML file, then file viewing is dependent on the original parsing tool being (1) present at navigation time, and (2) *stateless* in its execution.

For contrast, one example of a *stateful* parser is the uxtaf tool (Ladan, 2007), a parser and navigation shell for XBox 360™ disk partitions. It maintains an “environment” file that tracks, among other things, the current working directory of its custom shell between shell calls (e.g. the file records the new current working directory on calling “cd”). Simultaneous calls to extract files from separate directories are not supported by such a model, meaning DFXMLFS would need to support a global read lock, to be acquired when a file is read. Another stateful parser, hfsutils (Leslie, 1996), uses a single state file in the user’s home directory, making simultaneous access of multiple disks impossible.

Alternatively, implementing byte_runs elements to report file content locations makes later file viewing independent of the parsing tool. byte_runs elements also make the tool’s results more comparable with file system differential analysis (Nelson et al., 2014). However, they require a fairly complete understanding of a file system’s on-disk data structures, and if the DFXML generator is a tool extension, extensive understanding of the tool internals.

For the purposes of DFXMLFS, the objective level of DFXML generation is to report byte runs, but this is near the end of a simplified spectrum of “Feature completeness” of a generator. This is an approximate order of levels of completion for a generator:

1. Identifying directories and files.
2. Identifying directory and file timestamps.
3. Reporting file checksums for fixity.
4. Reporting byte runs.
5. Reporting other non-essential metadata.

These were chosen as generator development milestones due to the various types of approaches that can be taken for implementation (with examples of each approach given in a later section). All but the last can be used to fulfill “essential” metadata roles a full file system implementation must typically fulfill (especially inode data and data block pointers).

**Figure 4.**
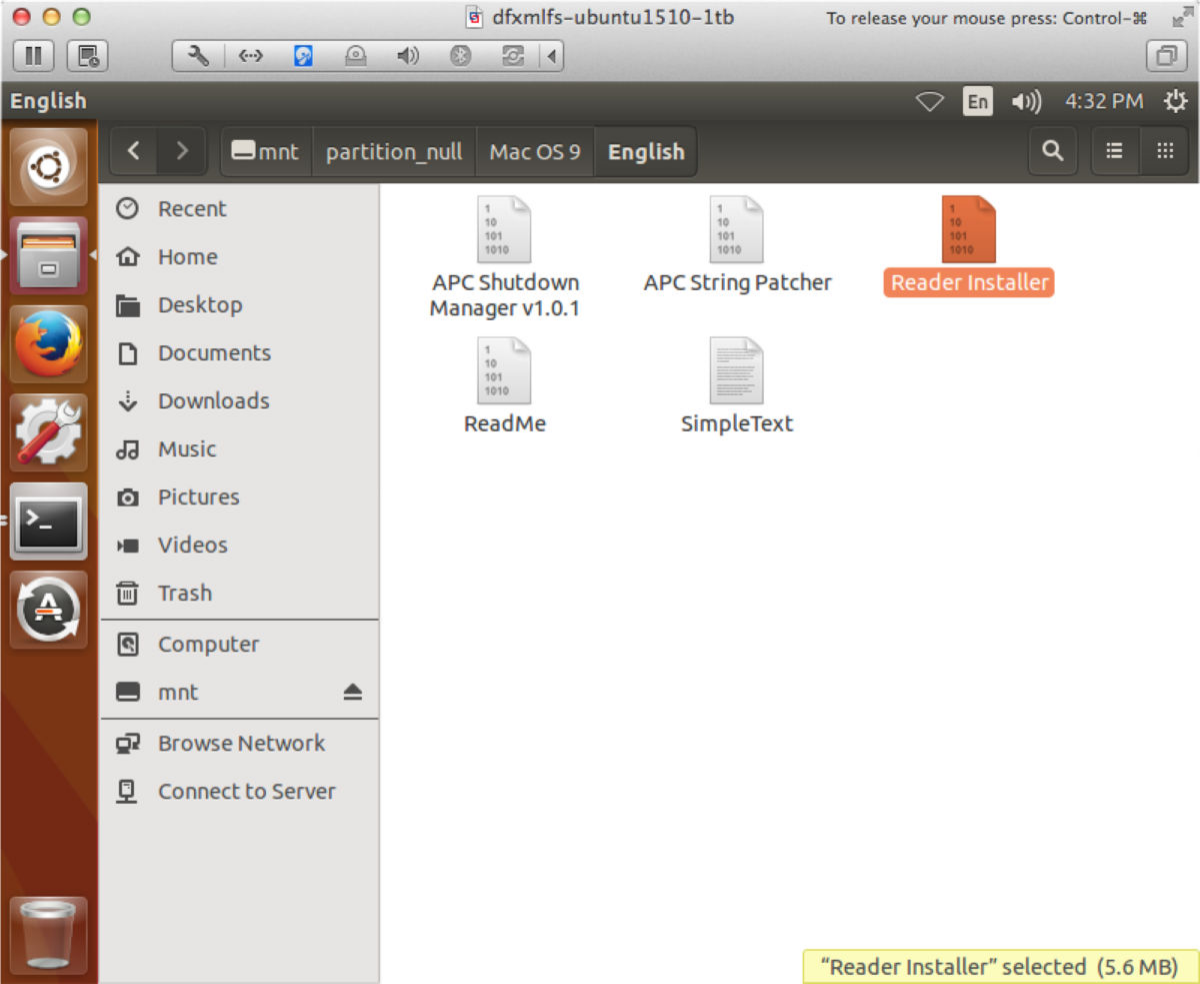
Screenshot of DFXMLFS mounting an HFS disk image with a supporting DFXML document. No HFS utility is used to mount the disk, but file content and metadata are available to the graphical file navigator—note there is sufficient information to populate a thumbnail from recognized file contents, and to report file size.

Figure 4 shows a working example of DFXMLFS, mounting the results of a tool that generates byte_runs elements, *hfs2dfxml* (Dietrich, 2015). Because the data block references are encoded in the XML, this textual representation of Apple HFS file systems can be mounted on a system without any HFS parsers present, including the *hfsutils* suite that originally generated the XML.

### Versus file-set approaches

Another alternative to using DFXML or DFXMLFS is to simply provide as parser output the set of all files the tool could find, perhaps packaged as a compressed archive. As an alternative to providing a simple file set, the DFXMLFS approach offers some advantages, including:

- Some timestamps, aside from modification time, cannot be preserved in an extracted file set. It could be important to an analysis to know what the original creation time of a file was, but that cannot be re-created for an extracted file, because the host operating system will overwrite that timestamp with the time of extraction—when the file was “created” on the host file system (Grier, 2011).
- A storage system that violates name uniqueness could cause files in an extracted set to be overwritten. DFXML provides a file metadata manifest that can detect name duplication.
- DFXML can be used to compare tool results at a finer metadata granularity than extracted files (Nelson et al., 2014), in part because some fields are difficult to preserve when extracted to a new host file system *(e.g.* rarely-implemented time stamps and extended metadata attributes that may not be supported in the content-presenting operating system).
- A polyglot storage system, such as a CD-ROM that presents two file systems for multiple operating systems while sharing data pointers (e.g. PC-Mac hybrid games from the late 1990’s), would be more cumbersome to report as a file set without use of hard links.

Deleted content offers a presentation-time challenge for both the file-set and DFXML approaches. DFXML provides deletion-analysis capabilities that a file-extracting tool could duplicate with a class of messages in its extraction log, but this induces another interface design to inspect the deleted content. If the end user wishes to see deleted content, both approaches would need to resolve issues with naming the deleted files in a way to avoid conflicts. A run-time option on DFXMLFS may offer more flexibility to the end user than having to rename files from a compressed archive.

## Potential Applications of the Framework

There are several usage scenarios that benefit from separating storage system parsing from navigation.

### Viewing file contents of obsolete storage

Digital curation is a practice that is likely to encounter storage systems that are no longer supported by current operating systems. For example, there are collections by artists who used early versions of Adobe Photoshop™ on Macintosh™ computers that only used the HFS file system (Dietrich and Adelstein, 2015). There are several challenges in curation at different levels of computing practice: Given the device, reading the bytes; given the bytes, parsing the file system; and given the files, viewing or migrating their content. The problem of interacting with bygone user-level applications is out of scope of this paper, but is handled by some others with software emulation (Woods, Lee, Stobbe, Liebetraut, and Rechert, 2015). The DFXMLFS approach leans closer to data annotation and migration.

Some curation exhibits only partially present computer storage contents to patrons (or students). One might not necessarily want to release file contents—e.g. file contents may require sanitization for privacy purposes—but it could be permissible for an exhibit to exhaustively list metadata. In this case, or if media are unavailable or yet un-processed, the XML can be mounted and a user can navigate the hierarchy, noting file names, and then requesting files that are key to their interests. DFXML could also be used to highlight subsets of a file system for other reasons, such as by showing files added or changed since a prior known state of the same disk, found by differential analysis (S. Garfinkel, Nelson, and Young, 2012).

### Low-bandwidth retrieval planning

For applications in which the communication bandwidth is extremely limited, obtaining all of the file contents of a storage device may be impractical or impossible, while metadata may be tenable. An example of such a domain is obtaining files from a device on another planet.

The communication path from Earth to the Mars Science Laboratory (Curiosity) has a portion that can only transmit in the hundreds of bits per second (Brat, Rungta, and Venet, 2013). The decision process on which of up to 300,000 rover files called “data products” to transmit back from the rover requires a prioritization queue system on the rover itself. Data products will have predetermined priorities at creation time, which can be changed by sending commands to the spacecraft. However, due to the relative positions of Earth, Mars, the rover, the orbiters, and the difference in time between an Earth day and a Martian day, human-in-the-loop planning and telecommunication asset scheduling sees latency of a full day or more.

A full manifest of data products may be requested from the rover in order for operators to determine the state of on-board memory. Moreover, a delta-manifest may be requested more frequently for correlating changes in memory state, *e.g.,* when items are created, deleted, or marked sent. These deltas are incorporated into a database consisting of all Earth knowledge of data products onboard the rover. Thus, the metadata for the rover’s data products is most often viewed as results of a database query.

Another way that manifest or delta-manifest of data products can be navigated is as a file system. A generator can construct DFXML from the rover’s delta-manifest, or a combination of these manifests, illustrating file system changes over time. DFXMLFS could then render the re-formatted manifest to a file browser, enabling operators and scientists to browse the data product manifest in a similar experience to walking the rover’s file system. Some operations staff may benefit from the alternative usability experience of browsing, searching, filtering, and making sense of the file system contents, using this view to update the data product delivery priority queue.

### Contemporary, but uncommon, storage

Some storage systems are not intended for general use. For example, the XBox™ and XBox 360™ game consoles used a custom variant of the FAT file system not used in any other computing systems (Nelson et al., 2014). That file system behaved much like FAT, but used custom data structures and a hard-coded partition management system, both of which required reverse engineering. The file system received a partial kernel implementation for FreeBSD (Ladan, 2007), but otherwise was mostly accessed by either game consoles or forensic tools. DFXMLFS normalizes access to such specialized storage systems, albeit in a read-only fashion.

### Parsing security

A forensic tool can pre-process a disk image to make the XML file, and then the XML is what is presented to the kernel Virtual File System layer. The original disk image is not presented to the VFS or kernel space. This provides a significant security benefit: if a storage system contains malicious constructs intended to attack vulnerable kernel code, a userspace program would not present the same attack surface. Additionally, if multiple parsers are used, an anti-forensic technique that attempts to evade an expected adversary’s parser may fail against alternate tools, and even highlight payload data with file system differencing.

### Presentation security

Content scrubbing and redaction is part of some digital archivist workflows. If an exhibit doesn’t intend to extract files from a disk image (e.g. because of wanting to preserve resource forks in an HFS image), a disk image can have sectors “white-listed,” using the byte run information to blank out everything but sectors essential for file contents, an example of partial disk imaging (Grier and Richard, 2015). In contrast, DFXML has been used by others to blacklist files (Woods et al., 2011; Woods et al., 2015).

## DFXML Generator Examples

There are several available open source DFXML generators. They demonstrate different approaches to parsing storage and normalizing the storage state, yielding different levels of completeness of essential file system metadata. On the sparser end of the spectrum, only directory and file names are reported—e.g. as derivable from a file manifest listing one file path per line. On the more complete end of the spectrum, content addresses are included for files, meaning no forensic tools need be required on the navigating computers. The order of the approaches here roughly decreases in effort required to generate DFXML, while simultaneously increasing dependence on a storage parsing tool to be present to let DFXMLFS provide file contents.

### Forensic tool APIs

Some forensic tools provide scripting or library support for using their parsing engine. The original DFXML paper (S. L. Garfinkel, 2009) introduced the tool Fiwalk as an extension to The SleuthKit, employing The Sleuthkit’s internal bindings to direct storage parsing and report the results as XML.

For digital forensic storage analysis tools that provide programmatic access to their parsing engines, it should be possible to produce DFXML given training or familiarity with the exposed APIs. For instance, the forensic tool EnCase provides a scripting engine with its own custom language. One user wrote a script in this language to use EnCase’s API to generate DFXML (Bourdon-Richard, 2014), including byte_runs elements.

### Forensic tool injection and extension

With some forensic tools, an API may not be provided for external code linking, yet it may be possible to extend software in any case. Nelson et al. (2014) extended two file system parsers^5^ to generate DFXML, by inserting generating functions and data structure support into the code bases.

If taking this approach, much can be learned from tool behaviors with debug print statements, such as what a tool believes are the addresses of directory tree data structures to name file references. Later, the logic making those debug print statements can be re-purposed to make DFXML instead—sufficiently complete to let DFXMLFS read the disk’s files without the (possibly customized) tool on the browsing system. However, care should be taken with this approach to not negatively affect the parsing routines as code is inserted.

### Forensic tool output parsing

Some storage forensic tools offer custom command-line navigation interfaces, producing output for every file. This kind of tool output can be parsed, converting un- or semistructured text output into DFXML, but requires implementing a custom “shell script” for what is a custom shell provided by the program. For instance, *hfs2dfxml* (Dietrich, 2015) scripts calls to the stateful shell provided by *hfsutils* (Leslie, 1996), parsing the text output with regular expressions.

This DFXML-generating approach may be a preferable alternative to modifying tool source code in some situations. However, if the tool does not provide addresses of data in any of its shell commands, then DFXMLFS cannot provide file contents.

### Userspace consumers

Some DFXML generators do not perform any storage parsing, and instead consume storage state as the operating system presents it to the user. The Python script walk_to_dfxml.py takes the data of the stat structure and formats it into DFXML with the Objects.py library. walk_to_dfxml.py also generates hashes by reading file contents, but has no access to block pointers, and thus cannot create byte_runs elements. Similarly, the *hashdeep* (Kornblum, 2008) family of tools written in C++ produces hashes for sets of files, relying on the kernel to handle storage parsing.

DFXML generated in this style, relying on the operating system to present storage contents, can be used to verify later that file contents are extracted consistently. However, beyond providing inode numbers and checksums, DFXML generated this way cannot further assist with file extraction if the storage parsing interface becomes absent. Such a DFXML file can be mounted and navigated, but attempts to read would receive “Not-implemented” error messages.

## Future Work

A library in the DFXML code base, Extractor.py, facilitates serving file contents using shell calls instead of byte runs. However, at the moment it is hard-coded to use The SleuthKit commands. Future development of Extractor.py, and an extension to the DFXML language, can standardize a <fileobject> child element that stores a filereading script. It is likely this kind of an interface, instead of byte runs, will be necessary in many cases for some file systems because of features like transparent compression and encryption. Other forensic languages or frameworks, such as Hansken (van Beek et al., 2015), include a notion of a forensic derivation chain similar to the forensic path of *bulk_extractor* (S. Garfinkel, 2013), where one can specify that a file must be decrypted, uncompressed, XOR’d, and have any other transformations applied. This type of recursive processing functionality does not presently exist in the DFXML libraries.

Another benefit comes from DFXMLFS separating storage parsing from interfacing.

DFXML created for one subject image and multiple independently-developed tools can have its contents verified, or flagged for further scrutiny, from differential analysis. One file system has received a DFXML-based storage meta-analysis. If forensic file sytem parser users contribute DFXML generators for their own subject media, storage forensic analysis as a whole benefits from getting cross-examinable results.

## Conclusion

The DFXML File System enables access to storage system contents without requiring one to have knowledge of implementing an operational file system, in kernel space or user space. It reduces the challenge of bridging the end user to an uncommon storage device; instead, our workflow calls for a simple parse of on-storage contents and the generation of a text file. A digital curator, and ultimately an end-user, should not require kernel-level file system implementation training to simply view the contents of antique or uncommon file systems. The creation of DFXML generators for non-contemporary file systems lowers the barrier to the curation and forensic communities to read their own instances of those storage formats.

Following the DFXMLFS workflow, only file system on-disk data structure knowledge is needed to navigate contents of an uncommon storage medium. DFXMLFS reduces the knowledge required to the more specialized and localized topic of the medium’s file systems, and the parsing can be done with analysis tools of varying implementation quality, from lightly-tested, experimental code that re-formats debugging print statements or current tool output, to full-fledged kernel modules. Formatting storage media contents into DFXML lets those contents be navigated like in their original setting, but separated in time and even space from the parsing process.

The forensic and curation communities benefit from having several generators available for any one file system type. Taken together, independent implementations of storage parsers improve understanding of individual storage systems, different file systems’ specifications, and of the practice of storage parser testing. These are all benefits of decoupling parsing and navigation, and further of using an in-common representation of file systems that differ vastly in on-disk organization. If an archivist or investigator is faced with a storage system their current operating system cannot mount, DFXMLFS presents a lighter coding path to reading the storage like any other file system of today.

## Figures and Tables

**Figure 1.**
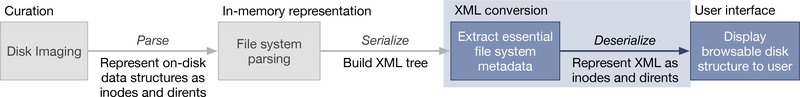
DFXML-based workflow: The disk image is parsed for the metadata in its inodes and dirents. These are serialized into an XML tree with essential file system metadata, and the result is later deserialized for user interaction. XML conversion and time-separated deserialization for display to the user are the core contribution of DFXMLFS.

**Figure 3.**
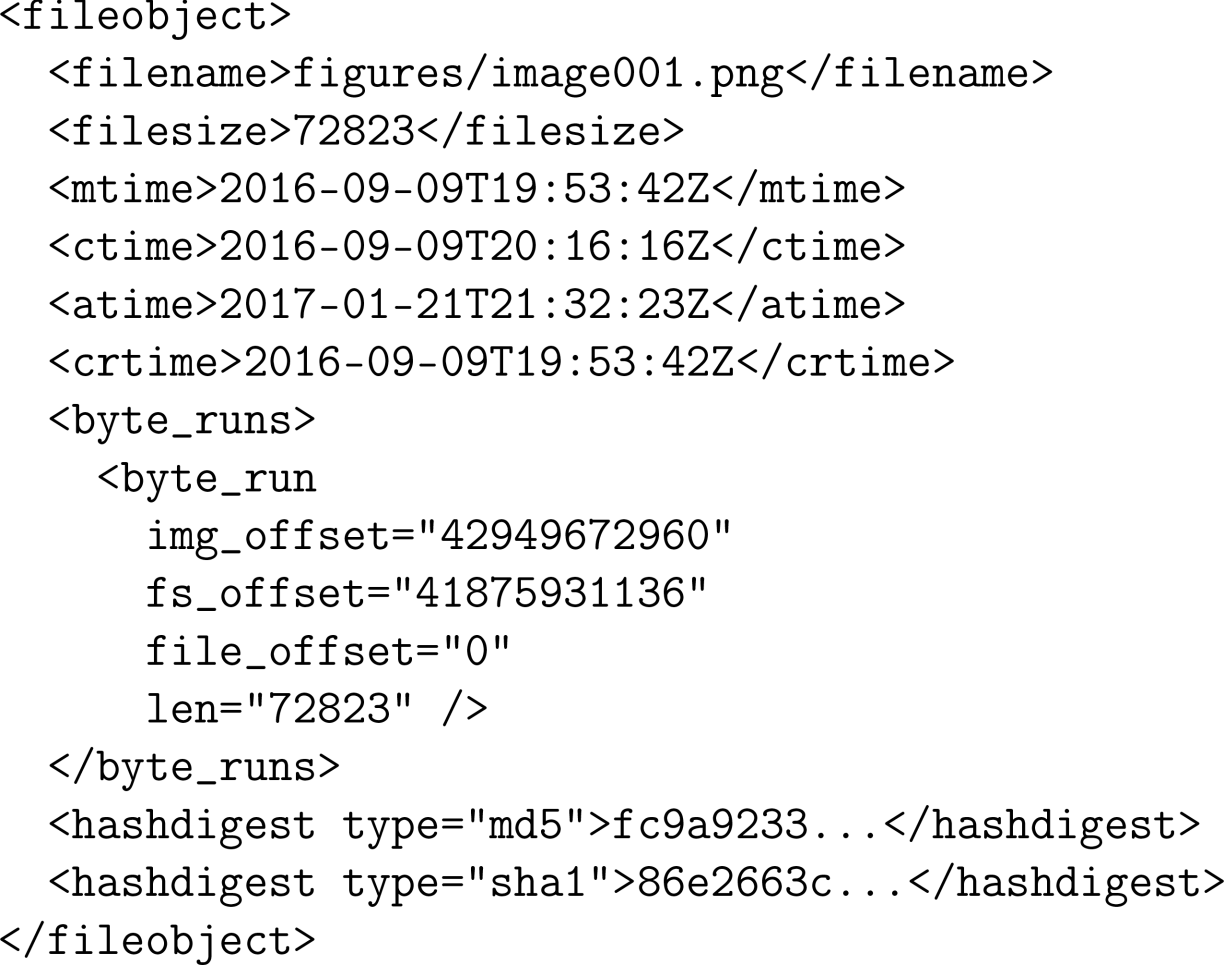
DFXML of image001.png from Figure 2. This illustrates output of a tool that provides data addresses. (Some content has been trimmed for print.)

**Table 1.** Strategies to enable file system interaction for uncommon storage media.

Strategy	Pros	Cons
Preserve *in situ* access, using original hardware	• Provides the original experience	• Entails maintaining the original devices • There may be issues with exporting data (*e.g.* no file system interface in game consoles)
Virtualize or emulate the original system	• Offers nearly original-device experience • Can be provided as-a-Service (Woods, Lee, Stobbe, Liebetraut, and Rechert, 2015)	• Requires extensive knowledge of original hardware • Efforts taken on one system don’t necessarily generalize
Implement or modernize a kernel module	• Allows a current host system using the chosen kernel to mount the storage device as normal	• Requires selecting a set of kernels to support • Requires working knowledge of the internals of the kernels chosen to receive development efforts • Suffers from effort fragmentation—kernel modules not guaranteed to be portable *(e.g.* BSD vs. Linux) • Run-time parsing faults cause bad browsing-user experience
Implement a FUSE file system	• Provides mostly same user experience as kernel module • Removes kernel development knowledge requirement	• Run-time parsing faults cause bad browsing-user experience
Extend, or design and implement, an independent forensic tool	• Tool can be written in the style of the implementer’s choice • Parsing faults seen by analyst, not necessarily browsing user	• Development freedom comes with the need for a user interface *(e.g.* custom command-line shell, custom GUI) • Non-standard storage interactions lead to fragmented user experiences

## Footnotes

1 Certain products may be identified in this document, but such identification doesn’t imply recommendation by the US National Institute of Standards and Technology or other agencies of the US Government, nor does it imply that the products identified are necessarily the best available for the purpose.

2 Durno (2016) provides many examples of tools with custom file system interfaces.

3 https://github.com/dfxml-working-group/dfxml_schema/blob/master/dfxml.xsd

4 https://github.com/simsong/dfxml/blob/master/python/Objects.py

5 The modifed versions of the two parsers, *uxtaf and py360*, can be found at: https://users.soe.ucsc.edu/~ajnelson/research/nelson_dfrws14/.
